# Cholinergic Basal Forebrain Connectivity to the Basolateral Amygdala Modulates Food Intake

**DOI:** 10.1523/ENEURO.0369-23.2024

**Published:** 2024-03-01

**Authors:** Joshua Ortiz-Guzman, Jessica L. Swanson, Evelyne K. Tantry, Mikhail Kochukov, Kevin Ung, Angela P. Addison, Snigdha Srivastava, Benjamin D. Belfort, Emily Ji, Sean W. Dooling, Sarah A. Chen, Qingchun Tong, Benjamin R. Arenkiel

**Affiliations:** ^1^Program in Developmental Biology, Baylor College of Medicine, Houston, Texas 77030; ^2^Department of Molecular and Human Genetics, Baylor College of Medicine, Houston, Texas 77030; ^3^Jan and Dan Duncan Neurological Research Institute at Texas Children’s Hospital, Houston, Texas 77030; ^4^Department of Neurobiology and Anatomy of McGovern Medical School, University of Texas Health Science Center at Houston, Houston, Texas 77030; ^5^Brown Foundation of Molecular Medicine for the Prevention of Human Diseases of McGovern Medical School, University of Texas Health Science Center at Houston, Houston, Texas 77030; ^6^Department of Neuroscience, Baylor College of Medicine, Houston, Texas 77030

**Keywords:** acetylcholine, basal forebrain, basolateral amygdala, circuits, feeding

## Abstract

Obesity results from excessive caloric input associated with overeating and presents a major public health challenge. The hypothalamus has received significant attention for its role in governing feeding behavior and body weight homeostasis. However, extrahypothalamic brain circuits also regulate appetite and consumption by altering sensory perception, motivation, and reward. We recently discovered a population of basal forebrain cholinergic (BFc) neurons that regulate appetite suppression. Through viral tracing methods in the mouse model, we found that BFc neurons densely innervate the basolateral amygdala (BLA), a limbic structure involved in motivated behaviors. Using channelrhodopsin-assisted circuit mapping, we identified cholinergic responses in BLA neurons following BFc circuit manipulations. Furthermore, in vivo acetylcholine sensor and genetically encoded calcium indicator imaging within the BLA (using GACh3 and GCaMP, respectively) revealed selective response patterns of activity during feeding. Finally, through optogenetic manipulations in vivo, we found that increased cholinergic signaling from the BFc to the BLA suppresses appetite and food intake. Together, these data support a model in which cholinergic signaling from the BFc to the BLA directly influences appetite and feeding behavior.

## Significance Statement

Feeding behavior has often been associated with homeostatic brain circuits in the hypothalamus. Here we identify a nonhomeostatic node of brain circuitry that functions to suppress feeding via cholinergic signaling from the basal forebrain. This circuitry may help explain how mood, motivation, reward, and aversion impacts feeding behavior.

## Introduction

Body weight homeostasis requires balancing energy expenditure with caloric intake. Energy imbalances are implicated in a wide range of diseases, including obesity-associated complications that contribute to mortality, such as heart disease, stroke, and cancer (Bouchard, 2008). It is broadly appreciated that feeding behavior is governed by the central nervous system (Morton et al., 2006). To date, a large body of research has focused on the hypothalamus, a major feeding center of the brain (Morton et al., 2014). However, numerous extrahypothalamic circuits sculpt and evaluate the sensory perception (Alonso-Alonso et al., 2015), quality, value, and availability of food (Johnson, 2013; Patel et al., 2019), acting together to directly influence appetite (McCrickerd and Forde, 2016; Riera et al., 2017). Notably, the diagonal band of Broca (DBB) within the basal forebrain is one such node with roles in sensory processing, attention, motivation, and more recently, appetite regulation (Herman et al., 2016; Patel et al., 2019). Ablation of cholinergic DBB neurons led to hyperphagia and obesity (Herman et al., 2016), and activation of excitatory DBB neurons downstream of cholinergic neurons led to hypophagia and starvation (Patel et al., 2019; Swanson et al., 2022). However, the DBB projects to numerous downstream nodes, and it is unknown which of these may drive the appetite suppression elicited by DBB activation.

ACh has been implicated in appetite suppression, and the acetylcholine receptor (AChR) agonist nicotine is a potent regulator of appetite (Picciotto et al., 2012). Moreover, muscarinic receptor knockouts reveal drastic changes in body weight (Gautam et al., 2006). Despite phenomenological evidence of ACh modulating appetite suppression, detailed neural circuit mechanisms underlying acetylcholine's appetite suppression remain largely unknown. The cholinergic DBB and its diverse projection system represent circuitry capable of directly synthesizing perceptual and homeostatic cues to modulate and/or directly govern feeding behavior. Given that the DBB is classically known to govern attention, motivation, and reward (Tashakori-Sabzevar and Ward, 2018) and that genetic ablation of the cholinergic neurons within the DBB results in obesity, we sought to identify downstream targets of the DBB that mediate appetite suppression.

Through genetically targeted anterograde tracing of DBB cholinergic neurons, we revealed that the DBB heavily innervates the basolateral amygdala (BLA). Although the amygdala has canonically been studied for its roles in fear and anxiety (Janak and Tye, 2015), more recently the amygdala has also been implicated in appetite regulation (Hajnal et al., 1998; Cai et al., 2014; Sun et al., 2015; Douglass et al., 2017; Kim et al., 2017; Calhoon et al., 2018; Hardaway et al., 2019; Pursey et al., 2019; García-García et al., 2020). Additionally, the BLA is known to assign valence to sensory stimuli to alter motivated actions (Beyeler et al., 2018). Thus, we reasoned that negative and positive associations formed via the BLA may act to promote or suppress appetitive behavior (Kim et al., 2017). Molecularly, there is a direct correlation between feeding and ACh release in the rodent BLA (Fitzgerald and Burton, 1981; Hajnal et al., 1998). Such evidence supports the BLA as a downstream candidate of the cholinergic DBB to modify feeding. To investigate this causative effect, we performed anterograde viral tracing and targeted electrophysiological recordings and revealed the BLA as a prominent downstream target of DBB cholinergic signaling. To test how DBB→BLA cholinergic signaling affects appetite control and sensory processing, we monitored amygdala activity and cholinergic release dynamics during food intake, which showed enhanced cholinergic release and BLA activation. Finally, using optogenetic and pharmacological approaches, we found that DBB ACh release in the amygdala is a strong driver of cholinergic DBB-mediated appetite suppression. Taken together, we show the BLA serves as a downstream effector of cholinergic DBB-mediated appetite suppression.

## Materials and Methods

### Experimental mouse lines

For all studies reported here, both male and female mice were included in analyses. Standard pellet mouse chow (Harlan, 2920X) was used for all experiments, and all animals were maintained on a normal 12 h light/dark cycle. *Chat-Cre* [B6;129S6-Chattm2(cre)Lowl/J] and *vGlut2-Cre* [Slc17a6tm2(cre)Lowl] mice were originally purchased and are available from Jackson Laboratory and have been previously described in Rossi et al. (2011) and Vong et al. (2011), respectively. PCR genotyping for Cre was done using the following primers (forward primer: 5′-GCATTTCTGGGGATTGCTTA-3′, reverse primer: 5′-GTCATCCTTAGCGCCGTAAA-3′).

### Stereotaxic injections and viral constructs

For all stereotaxic injections, mice were anesthetized in an induction box using 3% isoflurane and were maintained under anesthesia using vaporized isoflurane with O_2_. All injections were performed using a stereotaxic apparatus synced to Angle Two software for coordinate guidance. For anterograde tracing from the DBB, *Chat-Cre* mice (12 weeks old) were injected bilaterally into the DBB (ML, ±1.34 mm; AP, 1.10 mm; DV, −5.80 mm) with 500 nl per hemisphere of a conditional recombinant adeno-associated virus (rAAV) EF1α-DIO-Syn::mRuby2-WPRE-hGHpA, serotype DJ/8. Accurate targeting was identified by staining against ChAT in the DBB for post hoc identification of the injection site. For synaptophysin tracing from the BLA, *vGlut2-Cre* mice (8–12 weeks old) were injected bilaterally into the BLA (ML, ±3.35 mm; AP, −1.60 mm; DV, −4.90 mm) with 500 nl per hemisphere with a conditional rAAV-EF1α-DIO-Syn::EGFP-WPRE-hGHpA, serotype DJ/8.

### Fiber photometry feeding assay and analysis

vGlut2-Cre mice were injected in the DBB with rAAV-hSyn-DIO-GCaMP8 or rAAV-hSyn-DIO-GACh3.0 (as described above) and were bilaterally implanted with silica fiber optics (Thorlabs) situated over either a more anterior BLA (ML, ±3.05; AP, −0.70; DV, −5.15) or more posterior BLA (ML, ±3.35; AP, −1.60; DV, −5.30). The fiber optics were held in place by a headcap. Following surgery, mice were given at least 1 week for recovery and viral expression. All mice first underwent 5 d of 15 min habituation sessions in the behavioral chamber to acclimate them to the weight of a 0.48 nm, 200 µm core diameter fiber-optic cable (Doric Lenses). A fiber photometry system from Doric Lenses was utilized to simultaneously record from and stimulate both fluorescent vectors while mice remain freely moving. Both GCaMP8 and GACh3.0 were excited at 465 nm to record calcium binding and acetylcholine activity, respectively. The isosbestic points for both GCaMP8 and GACh3.0 are near 405 nm, allowing the emission generated by this channel to serve as a control for motion artifacts and noise. The photometry signal was coupled with video software (Doric Neuroscience Studio) to align feeding bouts with neural traces. The experimental timeline consists of four recorded sessions. Each session consisted of a 5 min acclimation period to the cable, and at least a 15 min feeding period where mice were presented premeasured pellet chow. The four sessions were paired, with each pair spanning the course of 3 d such that the first day measured baseline feeding activity (session 1), the second day mice were fasted overnight, and the third day measured fasted feeding activity (session 2). Paired sessions were conducted a week apart. Feeding sessions assessed activity during standard chow presentation (Harlan, 2920X).

Photometry traces were separated by mouse and recording session, without applying trial-to-trial or trial-averaged baseline subtraction or amplitude normalization. Thus, the values reported reflect isosbestic-subtracted d*F*/*F*. Mean and standard deviations for the entire recording were used for *z*-score calculation. *Z*-scored d*F*/*F* was calculated for the 3 s prior (baseline) and 1 s after initiation of each bite, which was determined by manual video scoring. Doric video capture software was used as a digital input during recording to precisely align fiber photometry recording with the video recording of feeding behavior. Once technical replicates of food bites were averaged for each individual mouse, biological replicates were averaged together to create a composite average. To calculate the average *z*-score feeding response as well as area under the curve (AUC), the average *z*-score response was calculated for 3 s prior (baseline) and 1 s postinitiation of feeding. Then, baseline was subtracted from the feeding response to generate a normalized feeding response across all bites. To simplify our data analysis, we excluded bites that were included during a feeding bout. Here, we define feeding bouts as instances of food intake where multiple bites overlapped within a 6 s period. AUC values were then compared using a one-way ANOVA test using Sidak's multiple comparisons with α value of 0.05. All analyses were performed in GraphPad Prism 8.

### CRACM and slice electrophysiology

For acute brain slice preparation, animals were anesthetized with isoflurane and perfused with cold artificial cerebrospinal fluid (ACSF) solution, pH 7.35, 310 mOsm/L, containing the following (in mM): 125 NaCl, 2.5 KCl, 1.25 NaH_2_PO_4_-H_2_O, 2 CaCl_2_, 1 MgCl_2_, 10 glucose, 25 NaHCO_3_. Brains were rapidly removed and transferred into sucrose-based cutting solution pH 7.35 containing the following (in mM): 87 NaCl, 2.5 KCl, 1.25 NaH_2_PO_4_-H_2_O, 0.5 CaCl_2_, 7 MgCl_2_-6H_2_O, 13 ascorbic acid, 75 sucrose, 10 glucose, 25 NaHCO_3_ equilibrated with 5% CO_2_/95% O_2_ gas mixture. We prepared 300-µm-thick coronal brain slices using a Leica VT1200 Vibratome and placed for at least 15 min at 37°C in 5% CO_2_/95% O_2_ bubbled ACSF solution. They were then gradually lowered to room temperature (RT; 25°C) and allowed to acclimate for at least 15 min before recording. For recording, slices were transferred into a recording chamber continuously perfused with ACSF at 1–2 ml/min at 25°C. Neurons were identified by transmitted light DIC (BX50WI, Olympus). Recordings were obtained using an Axon MultiClamp 700B amplifier digitized at 10 kHz (Axon Digidata 1440A). Recording electrodes (3–5 MΩ) were fabricated from borosilicate glass microcapillaries (outer diameter, 1.5 mm) with a micropipette puller (Sutter Instrument). The internal solution contained 110 mM K-gluconate, 10 mM KCl, 4 mM ATP-Mg, 0.5 mM GTP-Na, 10 mM phosphocreatine-di-Na, 1 mM EGTA, 10 mM HEPES, Biocytin 0.5%, pH was adjusted to 7.3 with KOH, the osmolarity – to 305 mOsm/L with K-gluconate. The principal neurons within the BLA were identified based on morphology, large cell capacitance (50–120 pF) and relatively low input resistance (70–250 MΩ). For a typical channelrhodopsin-assisted circuit mapping (CRACM) experiment, cells were voltage clamped at −70 mV for a several minutes to assess passive membrane properties and assure stability of the seal. The access resistance was monitored throughout experiment and was typically in a range of 10–20 MΩ, considered to be acceptable up to 30 MΩ. To assess inhibitory or excitatory nature evoked synaptic response, we switched patched cells to current-clamp mode and acquired light (10 ms pulse, 470 nm) induced responses with or without depolarizing current injections. GABAergic blocker bicuculline was continuously present in the bath to rule out inhibition through GABA release. To identify cholinergic nature of the response cells, we switched back in a voltage-clamp mode at −70 mV and applied a series of pharmacological treatments. Cells were recorded for a minimum of 10 sweeps for each pharmacological condition. Each sweep consisted of 2 s of recording, with an excitation duration of 10 ms of blue light (470 nm) exposure, with 30 s between each sweep. For evaluation of cholinergic connectivity onto BLA neurons, we used ACSF with the following final pharmacological concentrations in the slice chamber (in µM): 20 AP-5 (Tocris), 10 CNQX (Tocris), 20 bicuculline (Tocris), 10 atropine (Sigma), 1 TTX (Tocris), 0.5 4-AP (Tocris), and/or 0.5 mecamylamine (Tocris).

### Microscopy and immunohistochemistry

A minimum of 2 weeks was allowed for labeling in animals following viral injection. For processing, animals were deeply anaesthetized with isoflurane and were transcardially perfused with 1× PBS, followed by 10% neutral buffered formalin (NBF, Azer Scientific). Brains were dissected and postfixed in 10% NBF overnight at 4°C. Brains were cryoprotected in a 20% sucrose PBS solution at 4°C for 1 d, followed by a 30% sucrose PBS solution at 4°C for one more day. Brains were then embedded and frozen in OCT and stored at −80°C. Brains were sliced using a cryostat (Leica CM1860) in coronal sections 25–30 μm thick. For ChAT staining, 40 μm free-floating sections were blocked for 1 h at RT in 10% horse serum blocking solution in PBS-T (1× PBS, 0.5% Triton X-100, pH 7.35). Sections were then incubated with goat anti-ChAT primary antibody (1:500, Millipore, AB144P) overnight at 4°C in 10% horse serum blocking solution. Sections were then washed four times, 30 min each in PBS-T, and then incubated in secondary antibody (donkey anti-goat Alexa Fluor 488 or Alexa Fluor 555, Life Technologies) at a 1:1,000 dilution for 3 h at RT. Sections were then washed four times for 30 min each in PBS-T. All sections were mounted using DAPI Fluoromount-G (SouthernBiotech, 0100-20). Detection and imaging of fluorescent expression was performed using a Leica TCS SPE confocal microscope under a 10× or 20× objective.

### Tissue clearing

Following deep anesthesia with isoflurane, mice were intracardially perfused with 10 ml of 1× PBS and then 10 ml of 4% paraformaldehyde. Whole brains were extracted and placed in 4% paraformaldehyde overnight at 4°C, followed by an overnight wash in 1× PBS at 4°C. The brains were then incubated in 4% acrylamide hydrogel solution (5 ml of solution per brain) with gentle agitation overnight at 4°C. Next, the brains were transferred to a Logos Biosystems X-CLARITY Polymerization System (catalog #C20001) for tissue–hydrogel polymerization (settings: 37°C temperature, −90 kPa pressure, 3 h timer). After the polymerization cycle, the brains were washed in 1× PBS repeatedly until all residual hydrogel was removed. The brains were then briefly washed in Logos Biosystems electrophoretic tissue clearing solution (catalog #C13001) before being placed in a Logos Biosystems X-CLARITY Tissue Clearing System II (catalog #30001) for active electrophoretic lipid clearance (settings: 70 V voltage, 1 A current, 35°C temperature, 7 h timer, 100 pump speed). After completion of the cycle, the brains were washed repeatedly with 1× PBS for a total of 3 d. Finally, the brains were placed in Logos Biosystems Mounting Solution (refractive index matching solution, RI = 1.46 at 25°C, catalog #C13101) overnight at RT.

### Light sheet microscopy

A cleared mouse brain hemisphere (mid-sagittal cut) was affixed to an acrylic stage with super glue and submerged in 0.22 µm filtered 1% agarose. Once set, the sample-containing agarose was incubated in refractive index matching solution at RT for 3 d.

Samples were visualized using a Zeiss Lightsheet Z.1 side plane illumination microscope outfitted with a bespoke large bore sample chamber and a 5× dry objective. Resulting images were merged and stitched using Arivis Vision4D software. 3D reconstruction and figure generation were performed on Imaris image analysis software.

### In vivo feeding assays

*Chat-Cre* mice injected in the DBB with rAAV-EF1α-DIO-ChR2 (H134R)::EYFP-WPRE-hGHpA or rAAV-Ef1a-DIO-GFP-WPRE (as described above) were bilaterally implanted with 200 μm silica fiber-optic implants made in-house (Thorlabs, TS1249968); 230 μm ferrules (Precision Fiber Products, 2016 Macmillan Publishers Limited, MM-FER2007C-2300) and situated over the BLA (ML, ±3.35 mm; AP, −1.60 mm; DV, −4.70 mm). Fiber-optic implants were held in place by a cap made from adhesive cement (C&B Metabond Quick Adhesive Cement System, Parkell) for the initial base, and cross-linked with flash acrylic (Yates Motloid, 44115 and 44119) for the headcap. Mice were allowed at least 2 weeks for recovery and expression of the virus before assays were performed. All mice were first acclimated for 30 min in the behavioral chamber while tethered to a dual fiber-optic cord (Doric Lenses) attached to a 473 nm laser source (CrystaLaser CL-2005) prior to behavioral experimentation. After acclimation, mice were fasted overnight. In the morning, mice were presented with standard premeasured pellet chow, and food intake was recorded every 30 min for 2 h either under conditions of light stimulation (5 mW, 10 ms pulses, 20 Hz, 5 s trains, 30 s intervals) or without stimulation. Trials were randomized and conducted 1 week apart on the same animals. Independent samples *t* test used to compare “stim” and “no-stim” conditions between animals. As a control group, littermates were injected with GFP expressing virus as detailed above and implanted similarly to experimental mice. Comparisons between mock-stimulated and experimental cohorts used unpaired statistics for analysis.

### Cannula feeding assay

For pharmacology experiments, *Chat-Cre* mice (12 weeks old) were injected bilaterally into the DBB (ML, ±1.34 mm; AP, 1.10 mm; DV, −5.80 mm) with 500 nl per hemisphere of a conditional recombinant adeno-associated virus [rAAV AAV-Ef1a-FLEX-hChR2(H134R)-EYFP-WPRE-hGHpA] and then subsequently implanted with 0.22 NA, 200 µm core diameter fiber-optic implants (200 µm core with NA = 0.22, RWD *R*-FOC-L200C-22NA) into the DBB and drug cannulas (Plastics One Drug Cannula starter kit, 28 gauge single internal cannula, 26 gauge single guide cannula) situated over the BLA (ML, ±3.35 mm; AP, −1.60 mm; DV, −4.70 mm). Following surgery, mice were given 2 weeks for recovery and viral expression. All mice were acclimated to being attached to a 0.22 NA, 200 µm core diameter optogenetic cable (Doric Lenses) for 3 h. The mice underwent three 1 h feeding sessions, each session spaced a week apart to allow for recovery from fasting. Prior to each session, the mice were fasted overnight, weighed, and then placed into a behavioral chamber while attached to an optogenetic cable. Mice were given 1 h to acclimate to the chamber. During the next hour, mice were given premeasured standard chow pellets (Harlan,4 2920X) that were weighed at the end of the feeding assay to track mouse feeding. The food and mice were weighed immediately following the feeding paradigm. The first session measured mouse baseline appetite postfast. The second session introduced optogenetic stimulation (5 mW, 10 ms pulses, 20 Hz, 5 s trains, 30 s intervals) of the DBB. The third session incorporated both optogenetic DBB stimulation and a drug cocktail treatment of mecamylamine; MEC 20 mM plus atropine 1 mM into the BLA.

### Fos expression in sated and fasted animals

Wild-type animals were fasted overnight before being presented with standard pellet mouse chow for 1 h the following morning (fed group), while a second group of mice was fasted overnight but not presented with chow (fasted group). Mice were then immediately killed and perfused with PBS and 10% NBF. Brains were fixed overnight in 10% NBF before two overnight fixations in 20% and 30% sucrose/1× PBS solutions. Brains were frozen in OCT cutting compound and cryosectioned at 35 μm. Sections were then incubated for 1 h at RT in 10% horse serum blocking solution, made in 1× PBS-T (1× PBS, 0.5% Triton X-100, 0.1 mM CaCl_2_, pH 7.35). Sections were incubated with rabbit anti-c-Fos antibody (Calbiochem, PC38) overnight at 4°C at a 1:1,000 dilution in blocking solution. Sections were then washed four times for 30 min each in plain PBS-T. Sections were then incubated in secondary antibodies (Donkey anti-rabbit Alexa Fluor 594) at a 1:500 dilution for 3 h at RT and then washed four times for 30 min each in PBS-T. All sections were mounted using DAPI Fluoromount-G (SouthernBiotech, 0100-20). Detection of fluorescent expression was performed using a Leica TCS SPE confocal microscope under a 20× objective.

### OFA and EPM for anxiety measurements

*Chat-Cre* mice were injected with rAAV-Ef1a-DIO-ChR2::EYFP or rAAV-Ef1a-DIO-GFP-WPRE and implanted (as described above) to assess the effects of optogenetic activation of cholinergic terminals in the BLA on anxiety and fear. Prior to behavioral experimentation, animals were habituated to the experimenter and to the attachment of the fiber-optic cables for 3 min a day for 3 d. On the day of the experiment, animals were habituated to the room for at least 1 h prior to the initiation of behavioral experiments and allowed to recover for 1 min after attachment of the fiber optic prior to being placed in the open field apparatus (40 cm × 40 cm × 20 cm). During the behavioral test, mice were allowed to freely explore the apparatus during three 180 s phases: (1) a baseline phase before optogenetic stimulation (“pre-opto”), (2) a phase during optogenetic stimulation (“opto”), and (3) a phase after the optogenetic stimulation (“post-opto”). Animals were tracked, and measurements were recorded automatically using the ANY-maze software.

Adeno-associated viruses:

1. Cre-dependent synaptophysin: AAV-Ef1a-FLEX-Synaptophysin::mRuby2-WPRE-hGHpA
  1.1. Serotype: DJ8
  1.2. Titer: 4.06 × 10^12^ vg/ml
  1.3. Source Neuroconnectivity Core at the Jan and Dan Duncan Neurological Research Institute
2. Cre-dependent ChR2: AAV-Ef1a-FLEX-hChR2(H134R)-EYFP-WPRE-hGHpA
  2.1. Serotype:2/9
  2.2. Titer: 9.02 × 10^12^ vg/ml
  2.3. Source: Neuroconnectivity Core at the Jan and Dan Duncan Neurological Research Institute; subcloned from Addgene #40260
3. Cre-dependent GFP: AAV-Ef1a-FLEX-eGFP
  3.1. Serotype: DJ8
  3.2. 8.20189 × 10^12^ vg/ml
  3.3. Source: Neuroconnectivity Core at the Jan and Dan Duncan Neurological Research Institute; subcloned from Addgene #28304
4. Cre-dependent GCaMP: AAV-Syn-FLEX-GCaMP8s-WPRE-hGHpA
  4.1. Serotype: DJ8
  4.2. Titer: 3.53 × 10^12^ vg/ml
  4.3. Source: Neuroconnectivity Core at the Jan and Dan Duncan Neurological Research Institute; subcloned from Addgene #100839
5. Cre-dependent GACh3.0: rAAV-hSyn-DIO-GACh3.0
  5.1. Serotype: DJ8
  5.2. Titer: 7.93 × 10^12^ vg/ml
  5.3. Source: Neuroconnectivity Core at the Jan and Dan Duncan Neurological Research Institute; packaged from Addgene #121923

## Results

### DBB cholinergic neurons project to the BLA

**Figure 1.**
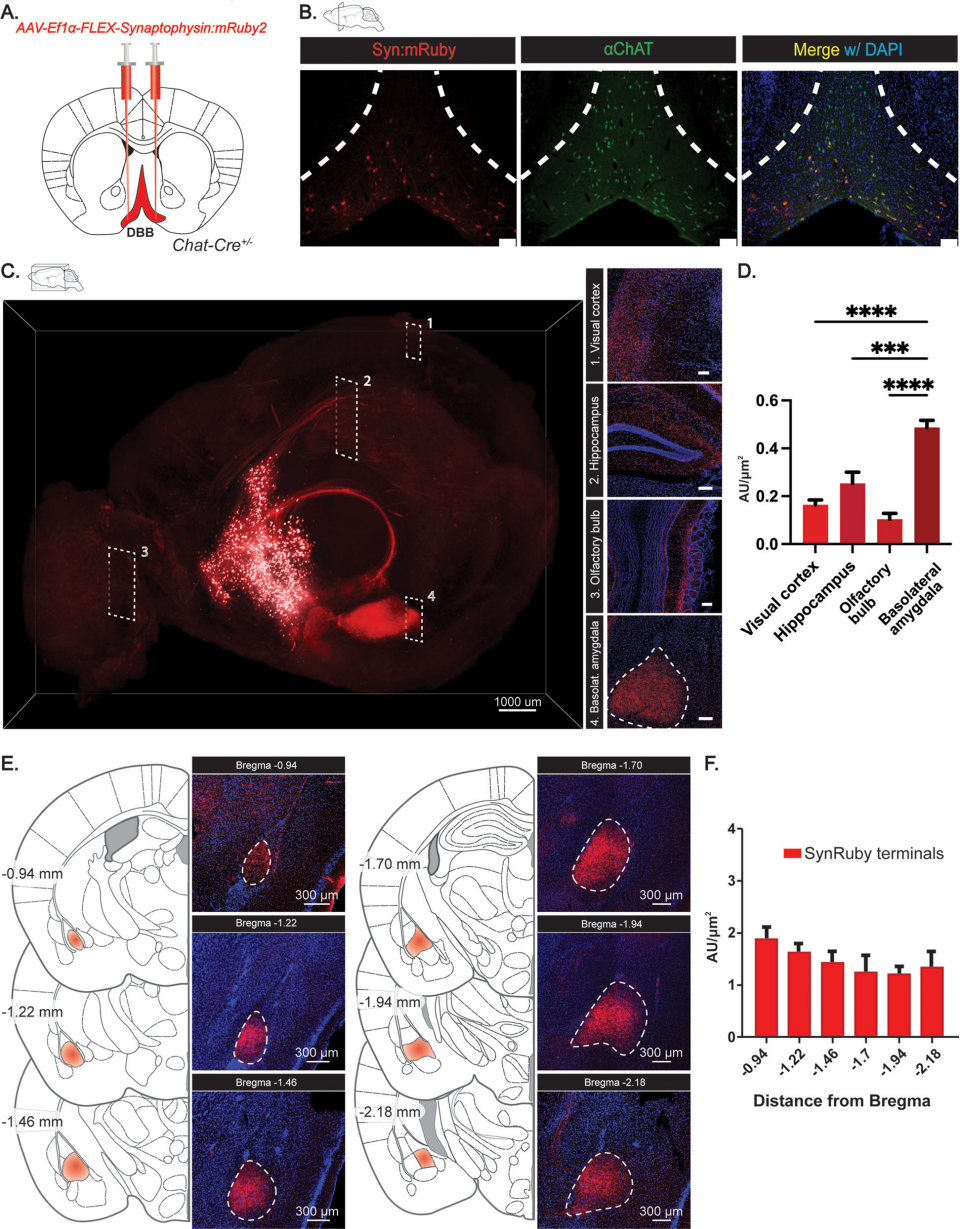
DBB cholinergic neurons send dense projections to the BLA. ***A***, Strategy for DIO-Syn::mRuby2 delivery to basal forebrain and mapping to the BLA. ***B***, Immunohistochemical confirmation of ChAT+ cell targeting in the DBB as detected by an anti-ChAT antibody and coexpression of the conditional AAV syn::mRuby2 reporter. ***C***, CLARITY image visualizing cholinergic projections from the DBB (scale bar, 1,000 µm). Representative images of the four most innervated brain regions downstream of cholinergic DBB neurons cholinergic terminals innervating the BLA from anterior to posterior (scale bar, 50 µm). ***D***, Quantification of cholinergic DBB projections to the visual cortex (0.1633 ± 0.120 AU), hippocampus (0.2533 ± 0.027 AU), olfactory bulb (0.1033 ± 0.015 AU), and BLA (0.4867 ± 0.018 AU), *n* = 3. ***E***, Representative images of cholinergic terminals innervating the BLA from anterior to posterior (scale bar, 50 µm). ***F***, Quantification of cholinergic DBB terminals along the A–P axis of the BLA, *n* = 7.

Cholinergic neurons of the DBB modulate appetite suppression (Galarce et al., 2010). To identify potential appetite-suppressing neurons downstream of the cholinergic basal forebrain, we targeted the DBB using conditional viral expression of the synaptically localized reporter Synaptophysin::mRuby2 (rAAV-Ef1a-DIO-Syn::mRuby2) in *Chat-Cre* mice (Fig. 1*A*,*B*). Three weeks postinjection, we observed multiple projection targets of DBB cholinergic neurons, including the hippocampus, olfactory bulb, median eminence, visual cortex, and the BLA (Fig. 1*C*). Of these, we noted that the BLA possessed a high density of ACh DBB terminals, anatomically implicating this structure as a downstream target (Fig. 1*D*). Additionally, using CLARITY (Kim et al., 2017) and light sheet imaging, we visualized DBB projections to the BLA in three dimensions, providing further evidence of robust connectivity between the DBB and the BLA, and that cholinergic terminals projecting to the amygdala selectively innervate the BLA but not other amygdalar substructures (Fig. 1*C*). Topographically distinct subregions within the BLA have been previously proposed to serve separate functional outputs for reward and aversion (McCrickerd and Forde, 2016). To query if subdomains within the amygdala may selectively receive DBB input, we quantified DBB ACh projection densities along the BLA's anterior/posterior axis and found that DBB cholinergic neurons exhibit broad projection patterns throughout the BLA, with heaviest labeling in the anterior domains (Fig. 1*E*,*F*). Since the BLA is known to possess functionally distinct subnuclei (McDonald, 1982; Kellis et al., 2020), these data suggest that DBB cholinergic signaling is relayed across all BLA subnuclei. However, the extent to which BLA subnuclei receive and respond to distinct DBB cholinergic input remains to be determined.

### DBB cholinergic neurons are functionally connected to the BLA

**Figure 2.**
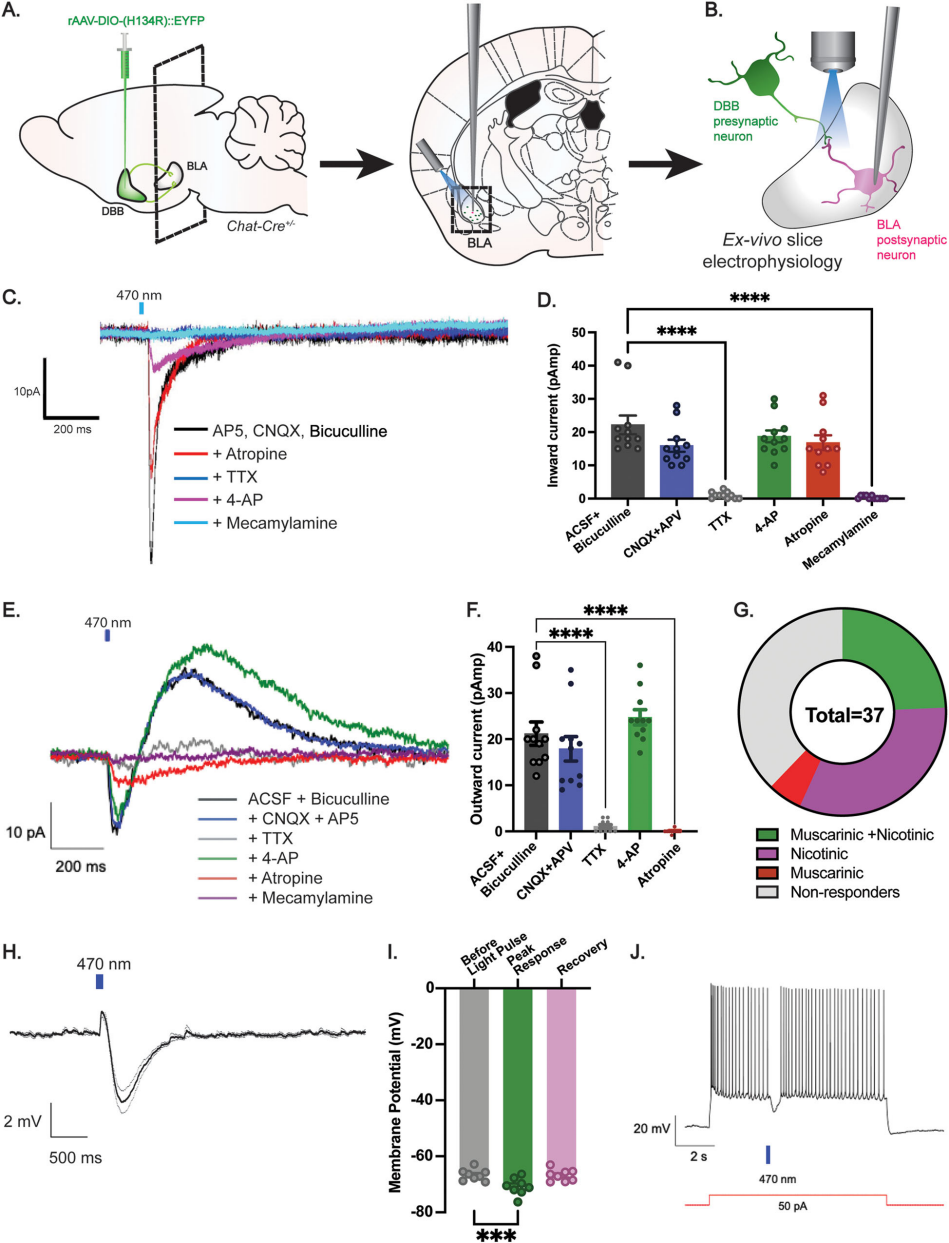
DBB cholinergic neurons are monosynaptically connected to BLA neurons. ***A***, Strategy for targeted ChR2 expression in the DBB, combined with slice electrophysiological recordings in the BLA. ***B***, Illustration of BLA-containing brain slice and recording site. ***C***, Example voltage-clamp recording from a BLA neuron after a 10 ms pulse of blue light, and with sequential pharmacological blockade of glutamate receptors (CNQX, APV), GABAergic transmission (bicuculline), muscarinic blocker (atropine), fast synaptic transmission (TTX/4-AP), and nicotinic receptors (mecamylamine). Cell demonstrates monosynaptic nicotinic responses. Trials included 20 s sweep with an intertrial interval of 1 min and repeated for five sweeps each for baseline. ***D***, Peak amplitudes (pA) of light-evoked inward current BLA neurons after optogenetic stimulation of DBB terminals in ACSF + bicuculline (22.18 ± 2.815 pA), CNQX + APV (15.91 ± 1.816 pA), TTX (1.000 ± 0.3015 pA), 4-AP (18.73 ± 1.784 pA), atropine (16.82 ± 2.235 pA), and mecamylamine (0.3636 ± 0.1521 pA). Error bars represent SEM. *n* = 11 cells, 4 mice. ***E***, Representative voltage-clamp recording from BLA neurons demonstrating both mecamylamine-sensitive inward current and outward atropine-sensitive response. After a 10 ms pulse of blue light, applied a sequential pharmacological blockade of GABAergic transmission (bicuculline), glutamate receptors (CNQX, APV), action potential-dependent synaptic transmission (TTX/4-AP), muscarinic blocker (atropine), and nicotinic receptor blocker (mecamylamine). Trials included 20 s sweep with an intertrial interval of 1 min and repeated for five sweeps each for baseline. Cell demonstrates monosynaptic nicotinic and muscarinic responses. ***F***, Peak amplitudes (pA) of light-evoked atropine-sensitive outward current in BLA neurons after optogenetic stimulation of DBB terminals in, ACSF + Bicuculline (21.18 ± 2.522), CNQX + APV (17.91 ± 2.651 pA), TTX (1.091 ± 0.3682 pA), 4-AP (24.73 ± 1.641 pA), and atropine (0.0909 ± 0.09091 pA). Error bars represent SEM. *n* = 11 cells, 4 mice. ***G***, Distribution of 37 recorded neural inputs between both muscarinic and nicotinic (24.3%), nicotinic (32.4%), muscarinic (5.4%), and nonresponders (37.8%). ***H***, Summary of recordings from BLA neurons in current-clamp mode. Trace shows an average of eight neurons and their baseline-adjusted hyperpolarization response (in mV) to blue light stimulation (10 ms). ***I***, Membrane voltage (mV) of BLA neurons before light pulse (−66.67 ± 0.718), peak hyperpolarization response after optogenetic stimulation (−70.81 ± 1.088), and during recovery post blue light stimulation (−66.79 ± 0.7508), all recordings done in the presence ACSF + Bicuculline. *n* = 8 cells, 3 mice. ***J***, Representative trace showing the effect of cholinergic inputs on principal BLA neuron firing due to a steady depolarizing current injection (50 pA). Light-induced activation of cholinergic terminals causes an IPSP and associated firing pause (ACSF + bicuculline).

Anterograde viral tracing data revealed anatomical connectivity between the DBB and the BLA (Fig. 1). However, anatomical tracing alone is not sufficient to determine whether associated brain regions are functionally connected. Thus, we used CRACM to optogenetically interrogate the synaptic connectivity between the DBB and BLA. Toward this, we stereotaxically targeted channelrhodopsin-2 via viral delivery [ChR2; rAAV-DIO-ChR2(H134R)::EYFP] to the DBB of *Chat-Cre* animals (Fig. 2*A*). Upon validating expression of ChR2+ fibers in the BLA, we next performed ex vivo whole-cell patch-clamp electrophysiological recordings from glutamatergic cells within the BLA (identified by morphology and electrophysiological properties; McDonald, 1982) while photostimulating ChR2-expressing DBB terminals and using pharmacological methods to interrogate the nature of the synaptic transmission between these two nodes (Fig. 2*B*).

Upon establishing whole-cell configuration under voltage clamp of glutamatergic BLA cells, ChR2-expressing cholinergic DBB terminals were stimulated with brief pulses of blue light. Upon photostimulation, we observed robust current in roughly 62% of recorded BLA cells. Additive wash-on of TTX and 4-AP indicated monosynaptic connectivity of DBB→BLA neurons responding to optogenetic stimulation. To isolate cholinergic responses, we used GABA and glutamate receptor antagonists (AP5, CNQX, and bicuculline) in all recording conditions. Since acetylcholine can bind to either ionotropic nicotinic or metabotropic muscarinic receptors, pharmacological antagonists for both nicotinic (mecamylamine) or muscarinic (atropine) receptors were used to determine the nature of DBB→BLA synapses. We identified some BLA neurons that displayed inward mecamylamine-sensitive nicotinic currents (Fig. 2*C*,*D*), some BLA neurons that displayed outward atropine-sensitive muscarinic currents (Fig. 2*E*,*F*), and some BLA neurons that displayed both nicotinic and muscarinic currents (Fig. 2*G*). Of the 37 BLA neurons recorded, 23/37 showed monosynaptic cholinergic responses. Of these monosynaptic responders, 12/23 cells showed exclusively nicotinic responses, 2/23 showed exclusively muscarinic responses, while 9/23 showed both nicotinic and muscarinic responses (Fig. 2*G*). Thus, cholinergic DBB neurons make functional, synaptic connections onto downstream BLA neurons, producing heterogeneous responses due to the differential expression of nicotinic and muscarinic acetylcholine receptors.

Due to the diverse downstream effects that ACh binding to its cognate receptor has on neurons, we next sought to further assess possible excitatory or inhibitory effects of DBB→BLA cholinergic transmission. Toward this, we tested light-evoked changes in membrane voltage in current-clamp mode. Once again, we recorded from excitatory BLA neurons while optogenetically activating cholinergic DBB terminals. To isolate excitatory responses, the GABA receptor antagonist bicuculline was used throughout recording. Eight out of 11 neurons recorded under such conditions showed light-induced hyperpolarization (2–6 mV from the resting voltage; Fig. 2*H*,*I*) and a brief (462 ± 92 ms) cessation of firing to injected current of just above the threshold (60–150 pA; Fig. 2*J*).

In sum, the cholinergic DBB is functionally connected to the BLA, and BLA cells receive cholinergic DBB input via both nicotinic and muscarinic acetylcholine receptors. Together, these data suggest that DBB cholinergic input may act on different BLA subpopulations to inhibit and/or activate specific populations of target cells to modulate appetite and feeding behavior.

### The BLA shows selective cholinergic signaling dynamics and activity patterns during food consumption

Anatomical tracing and electrophysiological recording data (Figs. 1, 2) support a functional relationship between the cholinergic DBB and the BLA. Recent studies highlight the BLA as a circuit node that impacts feeding, including cue-driven food intake (Galarce et al., 2010; Crouse et al., 2020; Servonnet et al., 2020), appetite suppression (Cai et al., 2014; Douglass et al., 2017; Kim et al., 2017; Hardaway et al., 2019), and high-fat diet preference (Parker et al., 2015; Hardaway et al., 2019; Ip et al., 2019). Since acetylcholine release has been observed in the amygdala in response to food intake or reward (Consolo et al., 1990; Hajnal et al., 1998; Crouse et al., 2020), we next questioned to what extent acetylcholine release in the BLA was correlated with feeding behavior.

**Figure 3.**
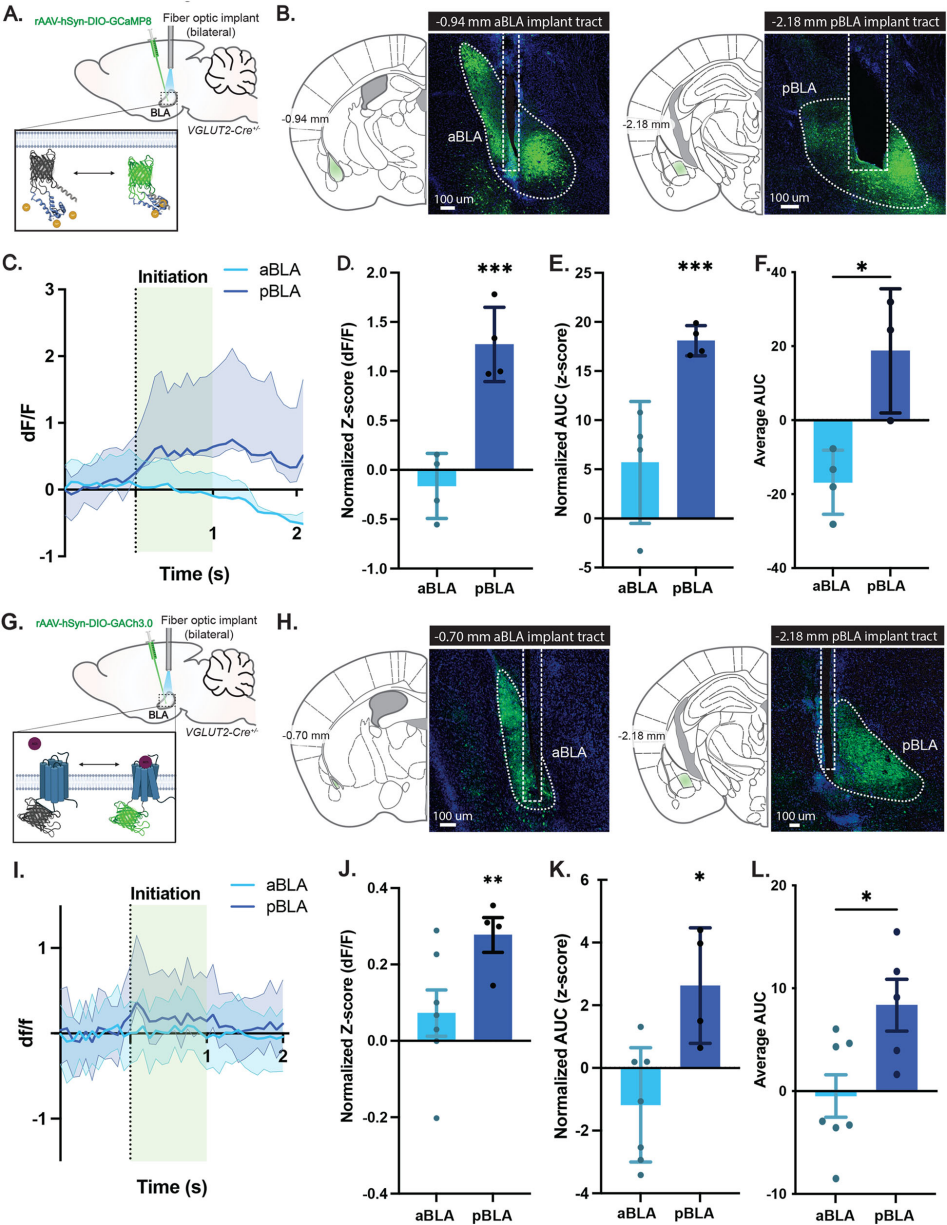
Feeding-induced increase in activity and cholinergic signaling in the BLA. ***A***, Diagram depicting injection and implant strategies for in vivo imaging using GCaMP and fiber photometry in the BLA. ***B***, Immunohistochemical confirmation of GCaMP expression in the BLA and fiber-optic implant placement. ***C***, Average of mean GCAMP d*F*/*F* traces for all animals. Shaded areas represent 95% confidence intervals. Expression along the A–P axis within the BLA during food consumption. ***D***, Mean food intake response of BLA neurons using fiber photometry. Error bars represent SEM. *n* = 4. Mean *z*-score d*F*/*F* food intake response: aBLA, *p* = 0.6943; pBLA, *p* = 0.0003. ***E***, Mean food intake response AUC of BLA neurons using fiber photometry. Food intake responses were baseline subtracted to normalize each value to its own baseline. Mean food intake response AUC was compared with baseline to determine statistical significance, which was calculated using a one-sample *t* test with the null hypothesis equal to 0. Error bars represent SEM. *n* = 4. Mean AUC of *z*-score d*F*/*F* food intake between aBLA and pBLA: aBLA, *p* = 0.0990; pBLA, *p* = 0.0001. ***F***, Mean food intake response AUC of BLA neurons using fiber photometry. Mean food intake responses were compared with each other using an unpaired *t* test. ***G***, Diagram depicting injection and implant strategies for in vivo imaging using GACh3.0 and fiber photometry in the BLA. ***H***, Immunohistochemical confirmation of GACh3.0 expression in the BLA and fiber-optic implant placement. ***I***, Average of mean GACh3.0 Δ*F*/*F* expression along the A–P axis within the BLA during food consumption. Shaded areas represent 95% confidence intervals. ***J***, Mean food intake response of BLA neurons using fiber photometry. Error bars represent SEM. *n* = 6. Mean *z*-score d*F*/*F* food intake response: aBLA, *p* = 0.0.3833; pBLA, *p* = 0.002. ***K***, Mean food intake response AUC of BLA neurons using fiber photometry. Food intake responses were baseline subtracted to normalize each value to its own baseline. Mean food intake response AUC was compared with baseline to determine statistical significance, which was calculated using a one-sample *t* test with the null hypothesis equal to 0. Error bars represent SEM. *n* = 4. Mean AUC of *z*-score d*F*/*F* food intake between aBLA and pBLA: aBLA, *p* = 0.2424; pBLA, *p* = 0.0184. ***L***, Average AUC quantification for anterior and posterior BLA during food intake.

To first evaluate the extent to which BLA target neurons respond to feeding signals, we sought to directly monitor the activity of BLA neurons during feeding behavior. Pyramidal excitatory neurons comprise 95% of all neurons within the BLA, and vGlut2 has been shown to be a ubiquitous marker for glutamatergic BLA neurons (Vong et al., 2011; Namburi et al., 2015). Leveraging this, we targeted a conditional genetically encoded calcium indicator, GCaMP8 (rAAV-Ef1a-DIO-GCaMP8-WPRE-pA), and a fiber-optic implant to two distinct BLA coordinates that received dense DBB input (anterior BLA defined as Bregma −0.70 to −1.46 mm; posterior BLA defined as Bregma −1.47 to −2.18 mm) either the anterior or posterior BLA of Slc17a6-Cre (vGlut2-Cre) mice to record the activity dynamics of topographically defined excitatory BLA neurons during feeding using fiber photometry in vivo (Fig. 3*A*,*B*). Injected and implanted mice were fasted overnight and then presented with food for 1 h, during which feeding behavior and BLA activity dynamics were recorded. Throughout the recording session, mice were video tracked for post hoc behavioral scoring to identify feeding bouts and align these time points with the fiber photometry recording data. GCaMP responses showed that posterior BLA activity significantly increased compared with baseline during food consumption (Fig. 3*C*,*D*; 1.272 ± 0.2045 AU; *p* = 0.0003). We also compared differential fluorescent reporter signals by measuring the response profiles via AUC comparisons between the anterior and posterior BLA groups during feeding (1 s post food intake). Comparisons to baseline revealed a significant increase in activity selectively in the posterior BLA during feeding (Fig. 3*E*; 18.09 ± 2.608 AU; *p* = 0.0001), with the posterior BLA showing significantly more GCaMP responsivity to feeding than the anterior BLA (Fig. 3*F*; 18.09 ± 0.7693; *p* = 0.0364). Thus, the BLA is activated in response to feeding initiation in a domain-selective manner.

Since acetylcholine has been shown to have appetite-suppressive effects (Hajnal et al., 1998; Herman et al., 2016; Thinnes and Klein, 2019), we next sought to directly monitor acetylcholine release within the BLA of *vGlut2-Cre* mice using the acetylcholine sensor GACh3.0 (rAAV-hSyn-DIO-GRAB-ACh3.0; Jing et al., 2020) during feeding using fiber photometry in vivo (Fig. 3*G*,*H*), recording differential GACh3.0 responses in both anterior and posterior domains of the BLA. Toward this, injected and implanted mice were fasted overnight and then presented with food for 1 h while cholinergic dynamics were recorded. Throughout the recording session, mice were video tracked for post hoc behavioral scoring to identify feeding bouts and align these time points with the fiber photometry recording data. Quantification of photometric recordings, including both the mean response and AUC, of the anterior and posterior BLA were compared before (3 s before food intake initiation) and during food intake initiation (the first 1 s of feeding). These data revealed that acetylcholine release increased significantly during feeding (Fig. 3*I*), with a reliable increase in GACh3.0 signal within 1 s following a bite of chow compared with baseline GACh3.0 levels (Fig. 3*J–L*; 0.2771 ± 0.09131 AU; *p* = 0.0037). Notably, the posterior amygdala responded more robustly to food intake than the anterior BLA (Fig. 3*J*; 0.2771 ± 0.068; *p* = 0.0020). As an initial analysis, we compared the fluorescent responses as AUC between the anterior and posterior BLA groups during feeding (1 s post initial consummatory action), compared with baseline prior to feeding. We observed a significant increase in baseline AUC values for the posterior BLA GACh3.0 activity (Fig. 3*K*; 0.2771 ± 0.09131; *p* = 0.0037) during food intake. Consistent with the GCaMP data, we observed a significant increase in average AUC scores for GACh3.0 in the BLA during feeding (Fig. 3*L*; 9.056 ± 2.730; *p* = 0.0214), suggesting increased signaling to the BLA is correlated with feeding behavior.

**Figure 4.**
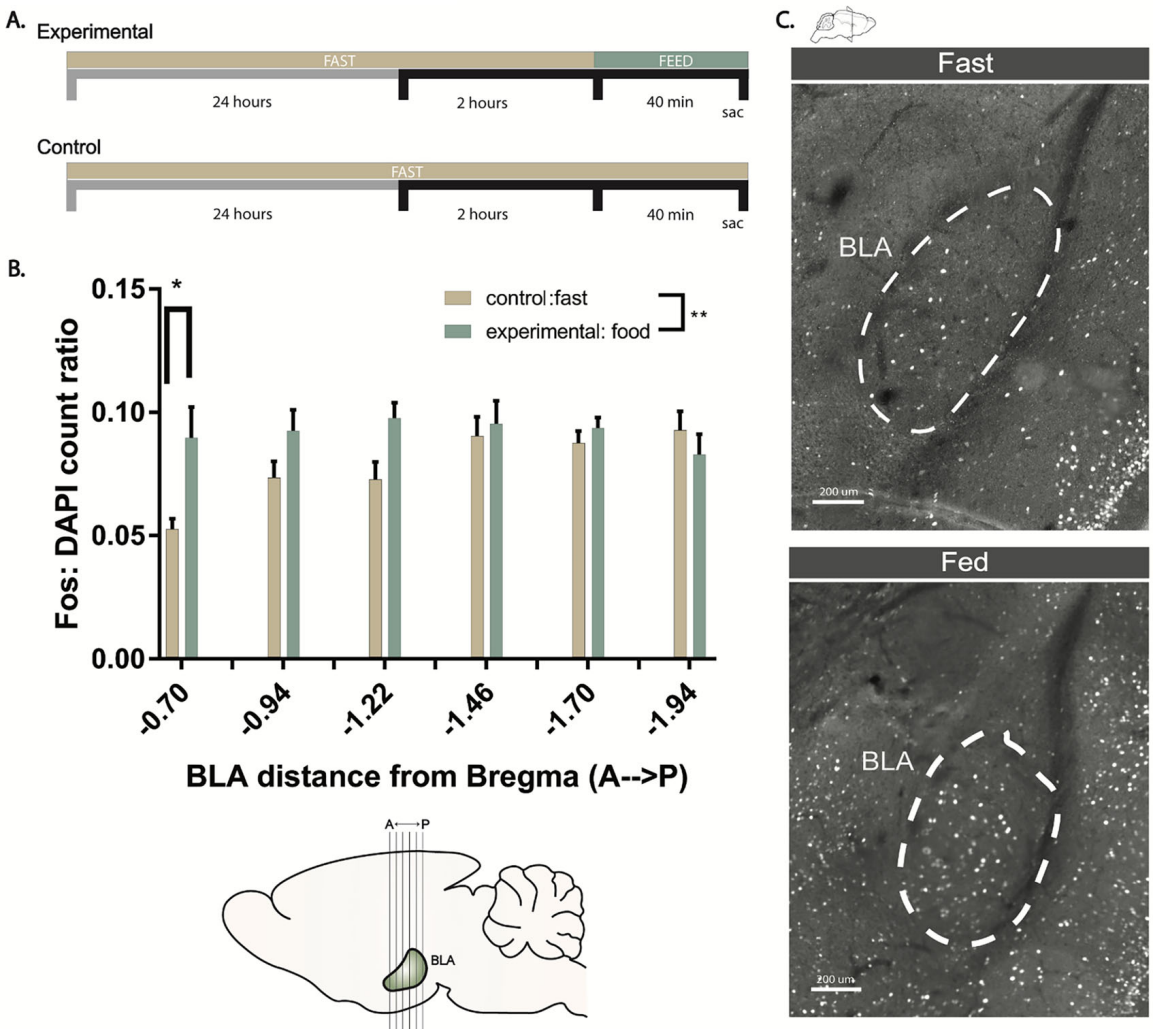
1 BLA activity is increased after feeding. ***A***, Diagram representing the feeding and behavioral timeline. ***B***, Quantitative measures of Fos expression along the A–P axis within the BLA. ***C***, Immunohistochemical confirmation of Fos+ expression in the BLA of Fed or Fasted animals (scale bar, 200 µm).

Alongside these experiments, we also independently evaluated Fos expression following a fasting and refeeding assay. Following a typical 24 h fasting period, experimental mice were given *ad libitum* access to food. Forty minutes later, animals were killed and tissues harvested, fixed, and processed for Fos expression (Fig. 4*A*). Subsequently, Fos expression was evaluated along the anterior–posterior axis of the BLA and quantified for differences against control animals that did not receive food (Fig. 4*B*,*C*). Consistent with real-time neuronal responses documented through in vivo imaging, Fos was upregulated in the BLA of animals after a fasting–refeeding period.

Taken together, these data show that both excitatory BLA neuron activity and BLA cholinergic release are dynamically increased during food consumption. Considering the BLA receives functional connections from the cholinergic DBB, these data support a model in which cholinergic DBB projections to the BLA may activate cells in the BLA to modulate appetite suppression.

### Activation of cholinergic DBB→BLA projections reduces food intake

**Figure 5.**
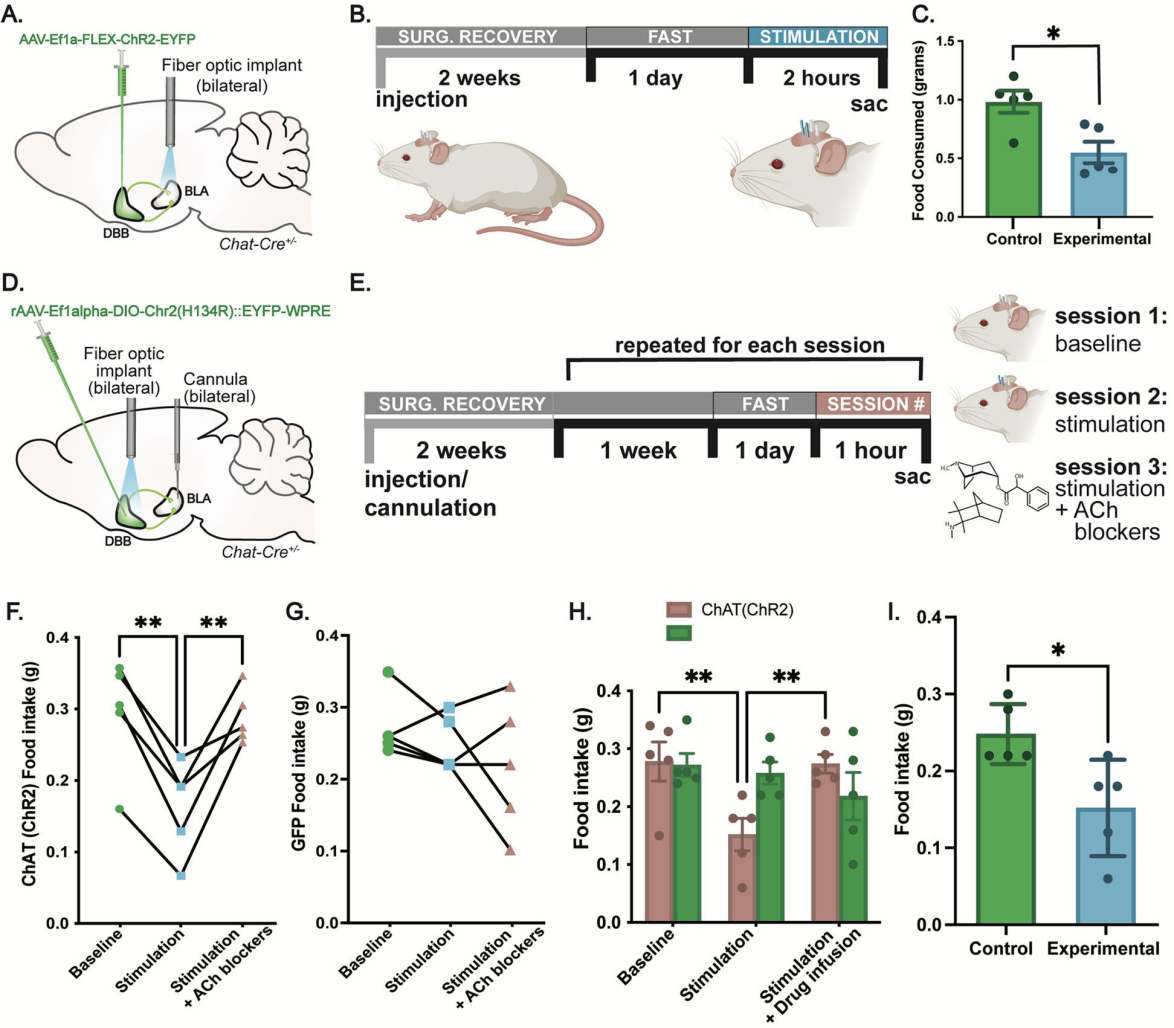
Optogenetic activation of DBB cholinergic terminals in the BLA induces appetite suppression. ***A***, Strategy for ChR2 expression in the DBB and fiber-optic implantation/stimulation in the BLA. ***B***, Diagram showing timeline and strategy for terminal field stimulation of DBB cholinergic fibers in the BLA and corresponding measurements of food intake. ***C***, Total food intake measured in grams for stimulated (experimental) versus GFP (control) animals. Statistical significance calculated using independent samples unpaired *t* test. Error bars represent SEM, *N* = 12, GFP controls, 0.982 ± 0.117 g; ChR2 animals, 0.550 ± 0.092 g; *p* = 0.0114. ***D***, Strategy for ChR2 expression in the DBB and fiber-optic implantation/stimulation in the DBB and drug cannulation in the BLA. ***E***, Diagram showing timeline and strategy of DBB cell body stimulation and drug cannulation of the BLA and corresponding measurements of food intake. ***F***, Total food intake measured in grams for individual ChR2 (experimental) mice across all conditions [baseline (nonstimulated), stimulated + vehicle, stimulated + drug]. Statistical significance calculated using repeated-measures two-way ANOVA with Bonferroni's multiple-comparisons test. Error bars represent SEM. *N* = 5. ChR2 animals: at baseline, 0.278 ± 0.034 g; at stimulation, 0.152 ± 0.028 g; at stimulation + drug infusion, 0.274 ± 0.016 g. Baseline versus stimulation, *p* = 0.0044; baseline versus stimulation + drug infusion, *p* ≥ 0.999; stimulation versus stimulation + drug infusion, *p* = 0.0057. ***G***, Total food intake measured in grams for individual GFP (control) animals across all conditions. Statistical significance calculated using repeated-measures two-way ANOVA with Bonferroni's multiple-comparisons test. Error bars represent SEM. *N* = 5. GFP controls: at baseline, 0.272 ± 0.020 g; at stimulation, 0.248 ± 0.017 g; at stimulation + drug infusion, 0.218 ± 0.041 g. Baseline versus stimulation, *p* ≥ 0.9999; baseline versus stimulation + drug infusion, *p* = 0.3602; stimulation versus stimulation + drug infusion, *p *≥ 0.9999. ***H***, Total food intake between ChR2 and control groups across all conditions. Statistical significance calculated using repeated-measures two-way ANOVA with Tukey’s multiple-comparisons test. Error bars represent SEM. *n* = 5. For ChR2 animals: baseline versus stimulation, *p* = 0.0034; baseline versus stimulation + drug infusion, *p* = 0.9916; stimulation versus stimulation + drug infusion, *p* = 0.0044. For GFP controls: baseline versus stimulation, *p* = 0.9022; baseline versus stimulation + drug infusion, *p* = 0.2459; stimulation versus stimulation + drug infusion, *p* = 0.4487. ***I***, Average food intake between ChR2 and control groups during stimulation. Statistical significance calculated using unpaired independent *t* test. Error bars represent SEM. For GFP controls: 0.248 ± 0.017 g. For ChR2 animals: 0.152 ± 0.028 g, *p* = 0.0196.

Previous studies revealed cholinergic DBB activation reduces food intake and that increased levels of ACh have been detected within the amygdala following food consumption (Hajnal et al., 1998; Crouse et al., 2020). Since we found that cholinergic DBB neurons are functionally connected to the BLA, that ACh release is detected in the BLA during food intake, and that the BLA itself is activated by food consumption, we next questioned if cholinergic DBB→BLA circuitry may directly modulate appetite suppression. Toward this goal, we targeted an AAV expressing conditional ChR2 [rAAV-Ef1a-DIO-ChR2 (H134R)::EYFP] into the DBB of *Chat-Cre* mice (Fig. 5*A*) and bilaterally implanted fiber optics over the BLA to photostimulate cholinergic DBB terminals. Control animals were injected with conditional GFP AAV (rAAV-Ef1a-DIO-GFP) and bilaterally implanted over the BLA. Since the posterior BLA was robustly activated by food intake as revealed via fiber photometry recordings (Fig. 3), we targeted fiber-optic stimulation implants to the more posterior domains of the BLA (ML, ±3.35; AP, −1.60; DV, −5.30). Following recovery, optogenetic mice were fasted overnight and subsequently photostimulated for 2 h while recording food intake (Fig. 5*B*). Consistent with a role for DBB→BLA connectivity in feeding, photostimulated ChR2-expressing mice showed a nearly 40% reduction in food consumption compared with stimulated GFP controls (Fig. 5*C*; *p* = 0.0114 using unpaired *t* test).

**Figure 6.**
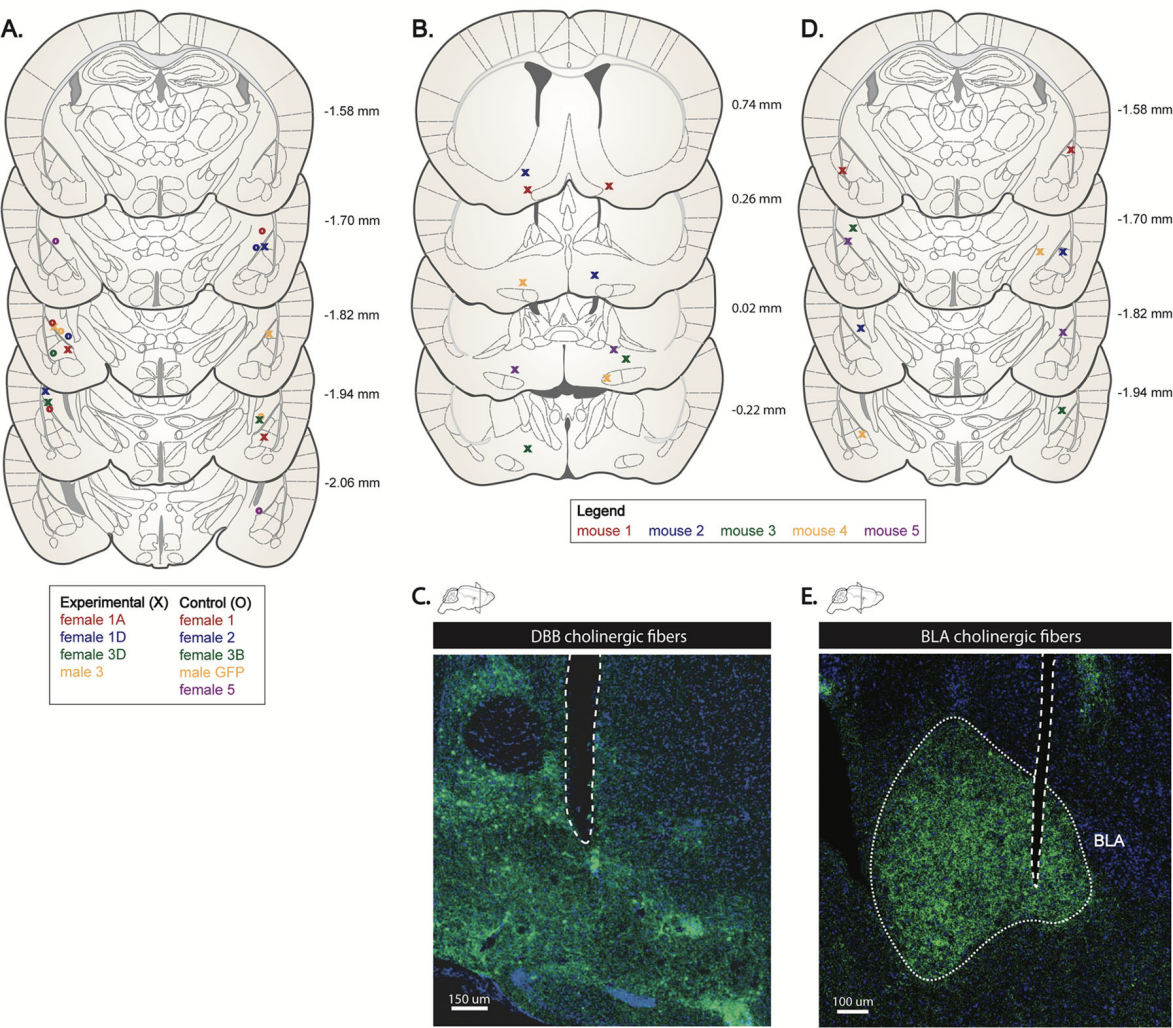
Fiber optic and cannula placement validation. ***A***, Fiber-optic implant site into the BLA for cholinergic terminal field stimulation in GFP versus ChR2 animals mapped onto the Allen Brain Atlas. ***B***, Fiber-optic implant site into the DBB for cell body in GFP versus ChR2 animals mapped onto the Allen Brain Atlas. ***C***, Immunohistochemistry depicting fiber-optic implant site into the DBB with ChR2+ expression for cell body stimulation in GFP versus ChR2 animals. ***D***, Drug cannula implant site into the BLA for stimulation in GFP versus ChR2 animals mapped onto the Allen Brain Atlas. ***E***, Drug cannula implant site into the BLA for drug cocktail infusion in GFP versus ChR2 animals mapped onto the Allen Brain Atlas.

**Figure 7.**
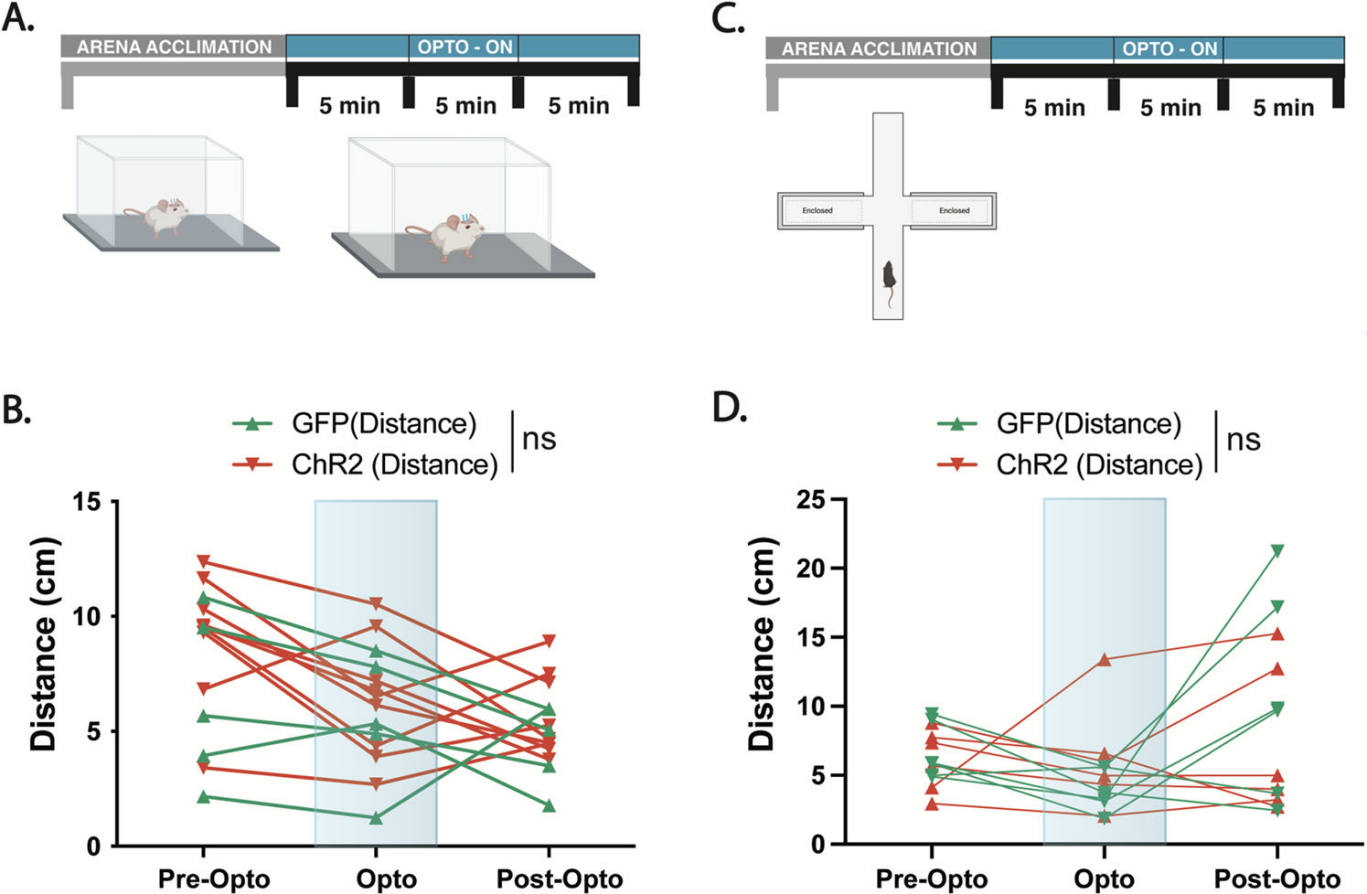
Cholinergic release in the amygdala does not alter locomotion. ***A***, Timeline for open field assay with stimulation paradigm. ***B***, Open field assay was separated into 3–5 min segments: prestimulation phase, stimulation phase, and poststimulation. Animals received blue light via an implanted fiber to the BLA. Distance traveled was recorded and graphed during all phases. ***C***, Study timeline during elevated plus maze assay with stimulation paradigm. ***D***, Elevated plus maze was separated into 3–5 min segments: prestimulation phase, stimulation phase, and poststimulation. Animals received blue light via an implanted fiber to the BLA. Distance traveled was recorded and graphed during all phases.

The BLA is one of several cholinergic DBB targets, all of which may modulate appetite simultaneously. To test the necessity of the BLA alone in cholinergic DBB appetite modulation, we paired optogenetic activation of the cholinergic DBB with pharmacological inhibition of the BLA. In other words, by optogenetically activating DBB cholinergic cell bodies while simultaneously inhibiting cholinergic receptors in the BLA, we selectively inhibited cholinergic signaling to the BLA while activating DBB cholinergic neurons and their non-BLA collaterals. Thus, if the BLA is the main effector of DBB cholinergic appetite suppression, inhibition of this node would occlude the appetite-suppressing effects of cholinergic DBB activation. Toward this, we targeted AAVs expressing either conditional ChR2-EYFP [rAAV-Ef1a-DIO-ChR2 (H134R)::EYFP] or conditional GFP (rAAV-Ef1a-DIO-GFP) to the DBB of *Chat-Cre* animals and implanted fiber optics bilaterally above the DBB to activate DBB cell bodies (Fig. 5*D*). At the same time, cannulas were bilaterally implanted over the posterior BLA to allow pharmacological infusion of cholinergic receptor antagonists during feeding assays. After recovery, doubly implanted mice were fasted overnight and the next day were provided access to food for 1 h. The first week, baseline food intake for fasted animals was recorded without any optogenetic or pharmacological manipulation. The second week, food intake was recorded while ChR2 and GFP mice underwent optogenetic stimulation with vehicle control infusion. As expected, optogenetic stimulation of DBB cell bodies in ChR2 mice with a vehicle control infusion resulted in robust decreased food intake compared with that in GFP controls during a 1 h feeding period, as previously described (Herman et al., 2016; Fig. 5*I*). The third week, mice underwent simultaneous optogenetic stimulation and infusion of broad spectrum AChR antagonists (a cocktail containing mecamylamine and atropine) into the BLA (Fig. 5*E*). Following AChR antagonist infusion into the BLA, optogenetic-driven decrease in food intake was abolished, resulting in normal feeding behavior to levels that were statistically similar to baseline, nonstimulated controls (Fig. 5*F*,*H*). Additionally, unstimulated GFP controls treated with AChR blockers showed no significant change in food intake, indicating that cholinergic inhibition of the BLA alone does not affect feeding behavior in fasted animals (Fig. 5*G*,*H*). These data indicate that the BLA is necessary for the decreased food intake effects of DBB cholinergic activation and that activation of DBB cholinergic projections to the BLA alone are sufficient for decreased food intake. All viral injection sites and fiber-optic implants were mapped post hoc to verify proper targeting (Fig. 6). Importantly, activation of this circuitry did not lead to anxiolytic or locomotor phenotypes, as assayed by open field tests and elevated plus maze (Fig. 7). Thus, selective activation of cholinergic DBB inputs in the posterior BLA resulted in decreased food intake. Together, these data suggest an appetite-suppressive effect of DBB→BLA circuitry and identify the BLA as a downstream effector of DBB cholinergic signaling in modifying food intake.

## Discussion

Feeding is a critical homeostatic mechanism regulated by multiple convergent neural circuits. While the hypothalamus has been extensively studied regarding homeostatic feeding, homeostatic and reward feeding mechanisms often overlap (Fitzgerald and Burton, 1981; Rossi et al., 2011). Here, we show that cholinergic DBB neurons project throughout the BLA, a major limbic node involved in emotional regulation and valence processing (Yang and Wang, 2017) Using viral tracing techniques, we identified the BLA as one of the cholinergic DBB's major downstream projection sites. To measure the electrophysiological effects of DBB ACh release, we recorded from neurons in the BLA while evoking synaptic release of ACh in ChR2+ fibers from cholinergic DBB neurons. We found that the majority of BLA neurons respond to DBB cholinergic input through fast-acting nicotinic receptors, leading to an excitatory response within BLA neurons. However, we also observed slower inhibitory responses via muscarinic signaling in a minor population of recorded BLA neurons. Interestingly, we also observed that BLA activity is directly linked with food consumption via GCaMP signaling and fiber photometry, in which feeding initiation resulted in increased activity of the posterior BLA. Likewise, we observed an increase in ACh levels within the amygdala after food acquisition, suggesting a tight relationship between food intake and food acquisition with release of ACh. Importantly, the overall net effect of ACh release within the amygdala during food intake resulted in decreased food consumption, and the appetite-suppressive effects of DBB cholinergic activation is dependent on the BLA. Altogether, these results support a model in which DBB cholinergic projections to the BLA modulate BLA neuron activity and drive appetite suppression.

The basal forebrain cholinergic system is associated with a wide range of behaviors, including attention, sensory processing, reward, and more recently appetite suppression (Herman et al., 2016). We have identified that DBB cholinergic neurons project to multiple brain regions, including the visual cortex, hippocampus, olfactory bulb, and, most robustly, BLA. Both the DBB and BLA play important roles in processing sensory stimuli (Golden et al., 2016; Patel et al., 2019; Sias et al., 2021; Swanson et al., 2022), and the BLA is a well-described emotional regulator that integrates external sensory information with valence. Additionally, the BLA is also known for its roles in fear and anxiety (Janak and Tye, 2015), and cholinergic signaling within the BLA affects memory consolidation (Picciotto et al., 2012). As appetite regulation is modulated by a combination of valence and sensory cues, recent studies have implicated the BLA as a node in appetite regulation (Kim et al., 2017; Beyeler et al., 2018). Here we provide further evidence that the DBB is a prominent source of cholinergic input to the BLA, and this connectivity is capable of modulating feeding behavior.

Using CRACM and electrophysiological approaches, we identified heterogeneous responses to cholinergic DBB input throughout the BLA. Notably, we found that DBB cholinergic signaling excites subsets of BLA neurons via nicotinic signaling but also inhibits other smaller populations of BLA neurons via muscarinic signaling. Furthermore, most cholinergic responsive neurons within the BLA show both types of responses, signaling through both nicotinic and muscarinic synapses. Thus, cholinergic input to the BLA may excite some appetite-suppressing cells, inhibit appetite-driving cells, and/or modulate other BLA cells in a more combinatorial or complex manner (Crouse et al., 2020). In fact, recent studies show that the homeostatic state of the animal can influence neural networks within the BLA (Ip et al., 2019). It is plausible that ACh may uniquely modify neural circuits to either excite or inhibit downstream targets in a dose- and/or state-dependent manner. Taken together, DBB cholinergic drive onto the BLA can result in modulating feeding behavior dynamics, yet the exact signaling mechanisms that govern such action remain to be elucidated (Pidoplichko et al., 2013; Unal et al., 2015; Jiang et al., 2016; Crouse et al., 2020; Kellis et al., 2020).

Interestingly, ACh release has recently been found to play an important role in the amygdala in the context of reward during a reward-retrieval task. Measuring cholinergic release via fiber photometry, we observed differential responses to food intake between discrete topological domains within the BLA (Kim et al., 2016). For example, cholinergic signaling in the posterior BLA showed the greatest increase in activation upon food intake. Likewise, ACh release upon feeding initiation was higher in the posterior BLA. This finding is corroborated by previous studies that show distinct topographically and genetically defined populations of BLA neurons, in which the anterior BLA drives appetite-promoting behaviors, while the posterior BLA drives appetite-suppressing behaviors (Kim et al., 2017, 2016). Other recent studies suggest a stratified hierarchy model of signal processing within the BLA, whereby excitatory cells show differential responses to aversive or appetitive stimuli relative to their anatomical location within the BLA (Kim et al., 2017, 2016; Beyeler et al., 2018). Collectively, these previous studies and our current results support that the amygdala is defined by topographically distinct and functionally different cellular responses to food intake.

Using optogenetics, we found that optogenetic activation of cholinergic DBB→BLA projections modulates appetite suppression. Notably, these observations were not confounded by alterations in locomotion or anxiety, as evaluated with EPM and OFA. Infusion of acetylcholine receptor antagonists into the BLA during DBB cell body optogenetic stimulation revealed that the BLA is necessary for the appetite suppression resulting from DBB activation. It is interesting to note that our electrophysiological findings suggest bidirectional modulation of the BLA by cholinergic DBB input, while our fiber photometry data reveal increased ACh release correlated with increased posterior BLA activity upon feeding initiation. While acetylcholine may have cell type-specific effects on BLA activity due to variability in acetylcholine receptor expression, since the majority of DBB→BLA synapses appear to signal via nicotinic receptors, this would lead to a net excitation of the BLA upon feeding, leading to an increase in BLA neuronal activity using population calcium imaging with fiber photometry. Thus, artificially driving ACh release in the BLA using optogenetic activation of DBB→BLA terminals may also work via nicotinic drive to induce potent appetite suppression. Perhaps acetylcholine is released at the start of a feeding bout in a form of anticipatory homeostasis to prevent overfeeding (Inglis et al., 1994; Hasselmo, 2006; Thinnes and Klein, 2019). This would explain the increased ACh release into the BLA, and subsequent BLA activation, in our fiber photometry recordings during food intake. As a result, activation of DBB→BLA neurons may mimic this state, suppressing feeding behavior in fasted animals.

In summary, we have uncovered a previously unknown circuit mechanism in which ACh release from the DBB to the BLA suppresses feeding behavior. It remains to be determined if subdomains in the BLA selectively express distinct cholinergic receptor subtypes and whether such populations may serve distinct functional roles in food intake and homeostasis. Additionally, loss-of-function studies in these specific cell populations may reveal the necessity of the BLA in modifying food intake. DBB→BLA cholinergic signaling provides a logical candidate brain circuit to be further investigated for its role in governing sensory processing and motivated behaviors such as feeding.

## Synthesis

Reviewing Editor: Lynda Erskine, University of Aberdeen Decisions are customarily a result of the Reviewing Editor and the peer reviewers coming together and discussing their recommendations until a consensus is reached. When revisions are invited, a fact-based synthesis statement explaining their decision and outlining what is needed to prepare a revision will be listed below. The following reviewer(s) agreed to reveal their identity: Mark Rossi.

Thank you for submitting your work to eNeuro. Whilst the reviewers were generally enthusiastic about the work and its contribution to the field, there were concerns about potential methodological issues and inconsistencies with the figures and related text descriptions. Below is a synthesis of the points raised by the reviewers. Most, if not all of these issues should be able to be addressed by changes to the text and figures.

General comments from the reviewers:

In this manuscript, the authors investigate the connections between the DBB and BLA using a series of viral-mediated tracing and functional circuit mapping techniques. They identify a cholinergic projection from DBB to BLA that is implicated in regulating food intake. Using fiber photometry, the authors demonstrate that neurons in the posterior BLA are especially responsive to food consumption, a time that corresponds to increased ACh release. They then use optogenetics to stimulate the DBB to BLA pathway, which suppresses food intake in hungry mice. Interestingly, this stimulation induced appetite suppression can be prevented by blocking ACh receptors within the BLA. Overall, the manuscript addresses an important question in the field; however, numerous inconsistencies within the figures and related text description should be addressed prior to publication.

This study investigates the connection between the DBB and BLA by characterizing the anatomical and synaptic properties of this projection. It also attempts to link the DBB-->BLA circuit to feeding. While I find the topic interesting and the authors have some nice approaches, the general presentation of the data, clarity of the studies, and methods is lacking.

Points to address:

1. Figure 1 E: it's difficult to assess location based on these zoomed in images and the hand drawn dotted lines of BLA seem subjective. Can you please provide more zoomed out images of BLA and if possible corresponding atlas images to make it more objective where the boundaries are?

2. Is there anything known about whether these Chat neurons co-release anything else besides acetylcholine?

3. Comments regarding Figure 2:

Fig. 2C-F: Please check the accuracy of the labels within the figure. 2C indicates GABAzine was used to block GABA receptors, but D shows bicuculline. In addition, the 4AP trace in panel C is ~5pA, but the quantification in panel D shows no value below ~18 pA. Similarly, the atropine trace in panel C is ~20 pA, but the largest current shown for the atropine condition in panel D is ~2 pA. A similar issue is observed between panels E and F. The amplitude of the atropine trace in E is clearly much smaller than bicuculline, CNQX/AP5, and 4AP traces but the bar graph indicates similar amplitude responses. What is the reason for these discrepancies? Clarification is needed in the description of this figure and the legend to link the example traces with the summary data.

I'm generally confused by Figure 2C-F and figure legend. Are these out of order? Also, if only 5 neurons had outward muscarinic currents, why are there more than 5 cells in Figure 2D? Figure 2 in general can use work on improving clarity of what is being presented.

Line 111: the authors describe inward and outward currents in Figure 2C, but it looks like it's entirely inward current in this image.

4. Presumably the BLA cells recorded were excitatory glutamatergic neurons rather than the less abundant interneurons? Is this correct and can the authors clarify in text whether they intentionally recorded form excitatory or inhibitory BLA neurons which can be distinguished based on membrane properties.

5. I'm not convinced that VGLUT2-cre is a good cre driver for BLA neurons (https://mouse.brain-map.org/experiment/show/73818754). People typically use EMX1-Cre as a driver for these neurons. VGLUT2 might be biased to LA or BA neurons so results here might be biased to a subset of BLA neurons and hence this should be made clear in the text by the authors.

6. Comments regarding Figure 3:

Figure 3 legend is not accurate. Panel A does not show a behavioral timeline. Panel C does not appear to show GCaMP expression across the AP axis as indicated in the legend but rather time series responses of BLA neurons. For panels C and I, how many trials/mice are being averaged? Error bars indicating the spread of the data should be included. Since the averaged values were calculated based on 3s after the initiation of a bite, the times series data should be extended beyond the currently shown 2s to allow the reader to evaluate the entire period that is being quantified in D-F and J-L.

What was the diameter of optic fiber implants used for photometry recordings? The images in Fig 3B suggest the diameter is <100um, but the track shown in the pBLA recording site looks substantially larger.

Figures 3B and 3H are too small to know where images are and lines drawn around BLA appear to be highly subjective and hand drawn.

The rectangles drawn for the "Fiber" implants are smaller in diameter than the 100um scale bar. I believe the authors used 200um core optic fibers so there's some discrepancy between the fiber tracks the authors indicate and the real diameter of the fibers. It makes trusting the proposed location of the fiber tracks difficult.

Based on coordinates in methods section, -0.7 would be extremely anterior BLA and -1.60 would be middle BLA. Posterior BLA extends back to -2.0 and further. Here again it's critical to show more zoomed out histology for evidence that placement is aBLA and pBLA.

I don't think it's appropriate to call experiments in Figure 3, aBLA or pBLA. For Figure 3H, what the authors call pBLA (-1.58) is too anterior to be confidently pBLA and is basically middle BLA.

7. Comments regarding Figure 4:

Fig. 4C: The Results section describes this being tested with a paired t-test (line 220), but since these are two different groups of animals, the pairing is inappropriate. Statistical significance should be determined using an independent samples t-test. Please also show individual data points for this panel. Statistical tests used for other panels in Figure 4 should be described.

In Figure 4D/E, what coordinates was the cannula targeted? The method section is lacking a lot of details on the cannula surgery, vendor, coordinates etc.

Regarding the feeding studies in 4F, I'm confused why fasted mice are eating so little (~0.3 g), which is assume is the total of the full 2 hour feeding assay based on the methods. Given the authors state they measured for every 30 minutes for 2 hours, the authors should plot the entire time course of the feeding assay as is standard in the field.

It's not clear what Figure 4I is analyzing. I assume it's the ChR2 comparing no stimulation session vs stimulation session, but this is not clear enough in the figure or legends.

8. Comments on methods:

Please elaborate on the slice electrophysiology methods. What internal solution was used? What voltage were cells held at for recordings?

Details for methods section on CRACM and slice electrophysiology is missing many details. How was slices prepared and what solutions were used.

Information about sources and titres of viruses used is missing.

Methods section on photometry is missing information. How was z-scoring actually performed (mean and standard deviation of what time periods were used) and how was dF/F calculated (what period of time was used as the baseline F).

Methods section is missing details about cannulas used for drug infusion experiments.

9. Please include the number of mice used in the legends for Figs 1 & 2.

10. Extended Data Figure 2: statistical test should be performed between before and after optogenetic stimulation on the locomotion assay. There appears to be a suppression consistent in all ChR2 mice when optogenetics is turned on.

11. Overall, the statement that this circuit "regulates feeding" is inappropriate as necessity studies were not performed. In other words, there are many brain areas that can be artificially turned on and disrupt ongoing behavior (feeding as well as other ongoing behaviors), but silencing of these neurons was not conducted to determine if that are involved in regulating feeding under certain conditions.
